# Evaluation of Metastasis-Associated Lung Adenocarcinoma Transcript 1 (MALAT 1) and H19 in Determining the Diagnosis and Severity of the Disease in Patients with Acute Pancreatitis

**DOI:** 10.5152/tjg.2025.24701

**Published:** 2025-11-06

**Authors:** Hafize Tugba Karahan, Alpaslan Tanoglu, Esra Guzel Tanoglu, Muhammed Said Gokce, Erdem Karahan

**Affiliations:** 1 Department of Internal Medicine, University of Health Sciences, Sancaktepe Şehit Prof. Dr. İlhan Varank Training and Research Hospital, İstanbul, Türkiye; 2 Department of Internal Medicine, Gastroenterology, Bahçeşehir University, İstanbul, Türkiye; 3 Department of Molecular Biology and Genetics, University of Health Sciences, İstanbul, Türkiye; 4 Department of Internal Medicine, University of Health Sciences, Sultan Abdulhamid Han Training and Research Hospital, İstanbul, Türkiye

**Keywords:** Acute pancreatitis, H19, long non-coding RNA, MALAT1

## Abstract

**Background/Aims:**

This current research targeted to assess whether long non-coding RNAs MALAT1 and H19, which are accepted to act a pivotal role in the progression of acute pancreatitis (AP), can be used as candidate biomarkers in addressing the diagnosis and severity of AP.

**Materials and Methods:**

Healthy volunteers who applied to the Internal Medicine outpatient clinic between October 2022 and June 2023 and patients who were hospitalized and treated for AP in the same period were enrolled in the research. The sociodemographic characteristics, biochemical values, and expression levels of MALAT1 and H19 of the study participants were compared.

**Results:**

There was no difference in terms of H19 level among the patient and healthy individuals (*P* = .619). However, a marked upregulation of MALAT1 expression was observed in cases of AP (*P* = .018). In addition, a range of biochemical markers showed notable disparities between the patient cohort and healthy individuals.

**Conclusion:**

According to the results of this current study, because the MALAT1 levels were significantly increased in AP cases, it could be postulated that MALAT1 can be used as a candidate biomarker in AP cases in order to diagnose the illness. However, MALAT1 and H19 did not correlate with the severity of the disease.

**Main Points**

- Acute pancreatitis (AP) is a major gastrointestinal disorder with global clinical significance, and its pathophysiology and diagnostic approaches continue to be actively investigated.
- The role of long non-coding RNAs in AP is being studied, and gray zones are existing.
- Metastasis-Associated Lung Adenocarcinoma Transcript 1 has emerged as a potential biomarker for the diagnosis of AP, offering promising implications for early detection and disease monitoring.

## Introduction

Acute pancreatitis (AP) is an acute inflammatory condition characterized by autodigestion of the pancreas, which is frequently encountered today and has significant mortality and morbidity rates. The diagnosis of AP is based on the presence of characteristic abdominal pain, biochemical evidence such as elevation of serum amylase or lipase levels to more than 3 times the upper limit of normal, and supportive findings obtained through imaging modalities. The presence of at least 2 out of these 3 criteria is sufficient to establish the diagnosis.^1^^-^^3^ Elucidating the etiology acts as an inevitable role in the correct management of diagnosis, treatment, and of course for clinical follow-up period.^4^ Etiology includes obstructive causes (stone, tumor, etc.), alcohol, metabolic causes (hypertriglyceridemia, hypercalcemia, etc.), trauma, hereditary causes, autoimmune causes, drugs and toxins, infections, vasculitides, anatomical or physiological anomalies. Despite all examinations, the underlying cause cannot be found in 15%-25% of patients, and it is called idiopathic AP.^4^^,5^ Despite all the developments in modern medicine and research to find therapeutic agents, there is no curative treatment other than intravenous fluid therapy and analgesic treatments.^6^^-^^10^ In the pathogenesis of AP, excessive stimulation of the pancreas combined with obstruction of the pancreatic duct leads to increased intraductal pressure and retrograde flow of active trypsin. This cascade triggers unregulated activation of trypsin within the acinar cells. As a result, premature enzymatic activation occurs inside the gland, initiating autodigestion of pancreatic tissue and promoting a localized inflammatory response.^11^ Conducting research to elucidate the pathophysiology of AP and identifying biomarkers that can predict disease severity are crucial steps toward reducing the morbidity and mortality associated with the condition.^4^^,^^12^

While previously the roles of long non-coding RNAs (lncRNA) in the pathogenesis of diseases were not clearly known, today’s studies have begun to reveal their molecular activities at the basis of diseases. It has been exhibited that lncRNAs participate in regulating the activity and localization of proteins. They also act in the organization and production of microRNAs and other RNAs.^13^^,^^14^ The lncRNAs are responsible for regulating gene activations at various levels. Impairment of this regulatory role leads to the development and progression of many diseases.^15^ Many studies have reported that lncRNA expression is irregular in various types of cancer and inflammatory conditions.^16^ The lncRNAs are found in various body parts such as the bloodstream, urine, and gastric fluid, and therefore, they can be used as biomarkers in various diseases.^17^

There are not enough clinical studies in the literature about the association between lncRNA expression and AP. This study aimed to assess whether lncRNAs MALAT1 and H19 can be used as biomarkers in the diagnosis of AP and in determining disease severity.

## Materials and Methods

First of all, ethical approval was obtained from Sancaktepe Şehit Prof. Dr. İlhan Varank Training and Research Ethics Committee dated September 24, 2022 and numbered E-46059653-020. During the same period, healthy volunteers who applied to the Internal Medicine outpatient clinic and patients with AP diagnosis who were hospitalized in the Internal Medicine ward were prospectively included in the study. After the participants were requested to sign a written information sheet about the study, 87 patients and 87 healthy individuals who volunteered to enroll in the research and signed the informed consent form were involved in the study. The diagnosis of AP is established when at least 2 of the following 3 criteria are met: a. Sudden onset of severe and sharp epigastric pain; b. Elevation of serum amylase or lipase levels to at least 3 times the upper limit of normal; c. Detection of radiological findings specific to AP on ultrasonography, d. computed tomography, or both. Criteria for inclusion in the study: AP patients who were admitted to the Internal Medicine clinic, healthy individuals ≥18 and ≤75 years old, cases with AP who volunteered to enroll in the study, cases without any known history of malignancy, persons without psychiatric disabilities who can give personal consent for the examination of file records. Exclusion criteria: individuals ≤18 years and ≥75 years of age, cases with chronic pancreatitis, pregnant women, breastfeeding mothers, individuals with any known history of malignancy, persons with psychiatric disabilities who cannot personally give consent for the examination of file records in the study, and patients with diseases other than AP that may increase the acute phase (patients with active infection, patients using antibiotics, patients diagnosed with acute renal failure, rheumatological diseases, etc.).

### RNA Isolation

RNA was isolated from the remaining blood samples after whole blood and routine blood tests were performed in the laboratory from the blood samples taken from the participants routinely within 24 hours. Total RNA was extracted from the same amounts of blood samples taken from patients and healthy volunteers using “One Step-RNA Reagent” (Biobasic, Canada) following the manufacturer’s protocol. The purity and concentration of RNA samples were assessed via spectrophotometric analysis using the NanoDrop ND-2000c instrument. (Thermo Fisher Scientific, Inc., Wilmington, DE).

### cDNA Synthesis and Quantitative Real–Time Polymerase Chain Reaction

A total of 300 µL RNA–cDNA reaction mixtures were prepared using the “OneScript® Plus cDNA Synthesis Kit” (ABM, Canada) in accordance with the manufacturer’s instructions. During the related procedures, the “BlasTaq 2X qPCR Master Mix” (Abm, Canada) kit was used. Base sequences of the primers are given in Table 1. Real-time polymerase chain reaction (qRT-PCR) reactions was performed as follows: 1 cycle of 95°C for 5 minutes, followed by 40 cycles of 95°C for 10 seconds, 60°C for 20 seconds, and 72°C for 25 seconds. The β-actin gene was used as the internal control. All reactions were performed in duplicate. Double delta-Ct analysis was utilized for qRT-PCR data interpretation.

### Biochemical Tests

The patients’ and health volunteer’s creatinine (mg/dL), amylase (U/L), LDL-C (mg/dL), lipase (U/L), glucose (mg/dL), urea (mg/dL), LDL-C (mg/dL), triglyceride (mg/dL), sodium (mEq/L), AST (U/L), albumin (g/dL), ALT (U/L), potassium (mEq/L), LDH (U/L), hemoglobin (g/dL), hematocrit (%), lymphocyte (10^3^/UL), platelet (10^3^/UL), neutrophil (10^3^/UL), leukocyte (WBC) (10^3^/UL), neutrophil/lymphocyte ratio, C-reactive protein (CRP) (mg/L), CRP/albumin ratio (CAR), platelet/lymphocyte ratio (PLR), procalcitonin (PCT) (µg/L) values were analyzed. Comparisons and statistical analyses were made between the groups with the data obtained.

### Statistical Analysis

In analyzing the data, mean and standard deviation, standard error, degrees of freedom, median, minimum and maximum values were used when statistics of continuous data were made, and frequency and percentage (%) values were used when defining categorical variables. The 95% CI of the difference in means between groups is given. Student’s *t-*test statistics were used to evaluate the difference in the means of 2 independent groups. Pearson Correlation test statistics were used to evaluate the relationship between continuous variables. The statistical significance level of the data was taken as *P* < .05. IBM SPSS 20 (IBM SPSS Corp.; Armonk, NY, USA) and MedCalc version 23.0.2 (MedCalc Software Ltd.; Ostend, Belgium) statistical package program were used to evaluate the data. In continuous measurements, parametric tests were used without normality testing due to compliance with the Central Limit Theorem.^18^ To determine the cut-off value for MALAT1 in predicting disease, ROC (Receiver Operating Characteristic) curve analysis was used. Significance was determined using AUC (Area Under the Curve) 95% CI values, Sensitivity, Specificity, Positive Likelihood Ratio, and Negative Likelihood Ratio statistics. Receiver Operating Characteristic curve analysis was performed using the MedCalc statistical package program.

## Results

A total of 174 people, 87 healthy controls and 87 AP patients, were enrolled in the study. While 42.5% of the participants were women, 57.5% were men. As a result, gender distribution was homogeneous in the patient and healthy groups (*P* = .99). The minimum age was 20 and the maximum age was 75, the mean age and standard deviation was 48.4 ± 15 and the median value was 49.

Oxygen requirement occurred in 6.8% of the patients and pleural effusion developed in 10.3%. Overall, 9.2% of AP cases had alcohol use and 12.6% had cigarette smoking. The average body mass index (BMI) of the patients was 28.85 ± 5.43 kg/m^2^. Requirement for admission to the intensive care unit (ICU) was present in 4.6%. The number of hospitalization days of the patients was determined as 8.9 ± 6.6. Etiologically, hypertriglyceridemia was detected in 11.5% of the patients, alcohol use in 3.4%, drug use in 2.2%, and biliary in 59.7%. In 22.9% of the patients, no etiological cause could be identified, and it was evaluated as idiopathic AP. Death due to AP occurred in 1.1% of the cases. Table 2 exhibits the clinical features of the participants and their distribution according to disease severity scores.

Demographic characteristics and biochemical values of patients and healthy volunteers are given in Table 3. Changes in H19 and MALAT1 cycle threshold values in patients and healthy groups are given in Table 4. H19 and MALAT1 difference tests between healthy and patient groups is given in Table 5. Assessment of relationships between H19, MALAT1 and other measurements in patients is given in Table 6. Assessment of H19 and MALAT1 in view of need for ICU admission is given in Table 7. With a cut-off of ≥3.02 determined for MALAT1, the test had a sensitivity of 70%, specificity of 55%, and a significant diagnostic power of AUC (95% CI): 0.60 (0.52 to 0.70); *P* = .027 in detecting the disease, while the positive predictive value was 65% and the negative predictive value was 62% (Figure 2).

**Figure 1.**
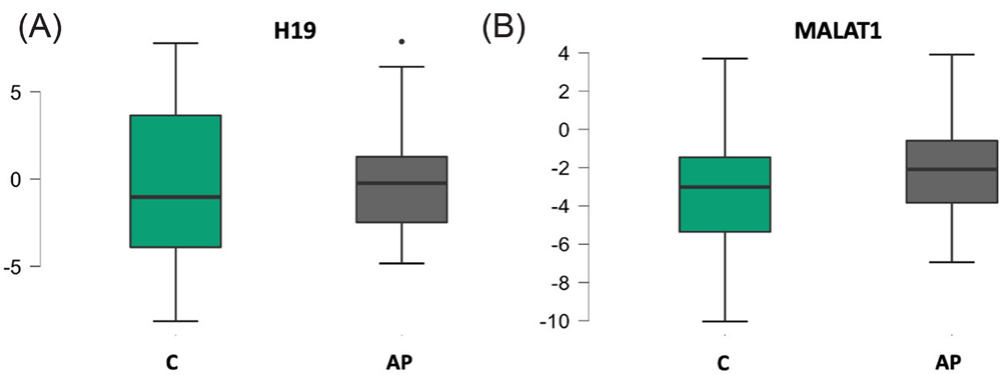
Expression levels of H19 and MALAT1. AP, acute pancreatitis; C, healthy controls.

**Figure 2.**
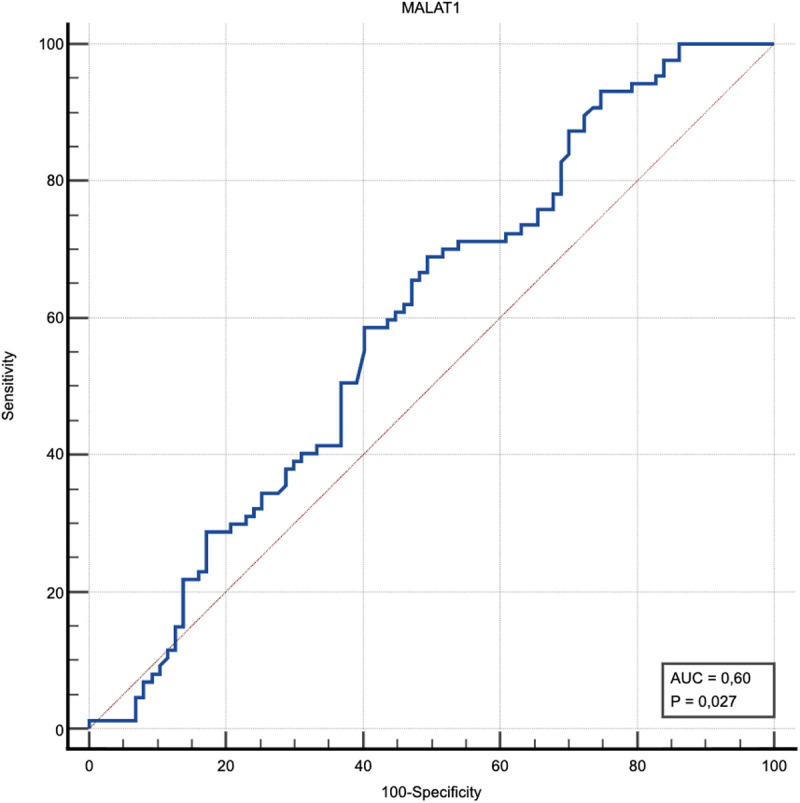
ROC curve of MALAT1 parameter in predicting acute pancreatitis.

In the present study, H19 expression levels did not differ significantly between the healthy control group and the AP group (*P* = .619). A statistically significant alteration in MALAT1 expression was observed between the healthy control group and the patient cohort (*P* = .018), and MALAT1 levels were found to be upregulated in the patient group (Figure 1). No significant relationship was found between H19 and MALAT1 and CRP, PCT, WBC, NLR, PLR, CAR, LACTATE, BMI, RANSON Admission Score, RANSON 48th Hour Score, BISAP Score, and length of hospitalization in patients. In terms of the need for ICU admission, a significant difference was found only in terms of the H19 value (*P* = .049).

## Discussion

In the experimental research accomplished by Sun et al^19^ in 2023, it was shown that lncRNA MALAT1 was highly expressed in rats that developed severe AP. Additionally, it has been shown that myocardial damage and inflammation in rats are alleviated when MALAT1 is knocked down.^19^ In the experimental study conducted by Li et al,^20^ it was exhibited that MALAT1 was overexpressed in AP. In the experimental study conducted by Lai et al,^21^ it was observed that MALAT1 was highly expressed in the pancreas and intestinal tissues of rats that were diagnosed with AP. Further investigation indicated that knockdown of MALAT1 could lessen pancreatic and intestinal injury, relieve inflammation, and alleviate the gastrointestinal motility disorders in rats.^21^ In the experimental investigation of Niu et al^22^ it was shown that MALAT1 has an important impact on AP development by triggering inflammation. Moreover, silencing of MALAT1 alleviates the damage to pancreatic tissue.^22^ In the study of Lee et al,^23^ when the pancreatic cancer tissues of rats were examined, it was noticed that MALAT1 was expressed at a higher level in those pancreatic tissues.^23^ In this current study, for the first time in the literature, MALAT1 expression was found to be significantly increased in the AP patient group, and this clinical finding was consistent with previous rat studies. Although no significant relationship was found between MALAT1 and H19 and parameters indicating the severity of AP, a relationship was found between H19 and the need for intensive care follow-up, and it would be appropriate to examine this issue with comprehensive studies.

In the experimental study of Song et al,^24^ it was shown that H19 improved the prognosis by inhibiting autophagy in rats that developed severe AP.^24^ In a randomized controlled study conducted in AP patients, lncRNA H19 levels were measured in serum samples taken from the patient and healthy control groups, and it was determined that H19 expression was increased in patients diagnosed with AP. Additionally, it has been observed that the H19 level increases as the severity of the disease increases.^25^ Although a sufficient number of AP patients and healthy controls were enrolled in the study, the application of sensitive and detailed inclusion and exclusion criteria may have caused no significant results in terms of H19.

Chronic pancreatitis may develop after recurrent AP.^26^ Approximately 5% of chronic pancreatitis patients develop pancreatic ductal adenocarcinoma. In the experimental study of Zhao et al^27^ lncRNAs were found to play a role in pancreatic fibrosis and pancreatic ductal adenocarcinoma development. The findings of this study indicate that lncRNAs may serve as promising biomarkers for the early diagnosis of pancreatic cancer in upcoming clinical applications.^27^ In the study conducted by Liu et al,^28^ it was shown that various lncRNA expression levels were upregulated in chronic pancreatitis and pancreatic cancer groups compared to healthy controls. This study emphasized that lncRNAs can be used as candidate biomarkers in the diagnosis of pancreatic cancer.^28^

There are studies showing that quercetin and emodin inhibit the inflammatory response by targeting lnc-RNA in the treatment of AP and significantly reduce mortality in rats with severe AP. However, more studies are needed on this subject.^29^

Despite its contributions, the study has inherent limitations that may affect the generalizability of the results. First of all, the study population was relatively small. Secondly, this current study was a single-centered research study. Despite these limitations, the results contain significant findings and may contribute to the understanding of molecular gray areas in AP.

In conclusion, to the best of current knowledge, this is the first time in the literature where MALAT1 and H19 expression levels were simultaneously used as candidate biomarkers for the determination and severity of AP in a prospective clinical study. Long non-coding RNAs, and especially MALAT1 expression levels, can be used as candidate biomarkers in diagnosing the disease in cases of AP. The CAR and NLR ratios, along with Ranson and BISAP scores, can be used to determine the severity of AP. In order to base these predictions on solid foundations, it is of great importance to plan and conduct experimental and clinical studies in correlation.

## Figures and Tables

**Table 1.** Base Sequences of the Primers Used in the Study

Genes	5′→3′
*MALAT1-F*	GAAGGAAGGAGCGCTAACGA
*MALAT1-R*	TACCAACCACTCGCTTTCCC
*H19-F*	CACTGGCCTCCAGAGCCCGT
*H19-R*	CGTCTTGGCCTTCGGCAGCTG
*Beta-actin-F*	GCCTCGCCTTTGCCGATC
*Beta-actin-R*	CCCACGATGGAGGGGAAG

**Table 2.** Clinical Characteristics and Severity Scores of Patients

Clinical Characteristic	Number of Patients	Rate (%)
Oxygen needs	5	6.8
Pleural effusion	9	10.3
Need for ICU hospitalization	4	4.6
Etiology of acute pancreatitis		
Hypertriglyceridemia	10	11.5
Alcohol	3	3.4
Biliary	52	59.7
Drug	2	2.2
Idiopathic	20	22.9
Scores and clinical features used to determine the severity of acute pancreatitis		
Number of days of hospitalization (mean ± SD)	8.9 ± 6.6	6 (2-61)
BMI kg/m^2^ (mean ± SD)	28.85 ± 5.43	27.7 (19-46)
RANSON at Hospitalization (mean ± SD)	1.23 ± 0.95	1 (0-4)
RANSON 48th hour score (mean ± SD)	1.92 ± 1.01	2 (0-6)
BISAP Score (mean ± SD)	0.51 ± 0.23	0 (0-4)

Student’s *t*-test.

ICU, intensive care unit; BMI, body mass index; SD, standart deviation.

**Table 3.** Comparison of Patients and Healthy Volunteers in Terms of Demographic Characteristics and Biochemical Values

Parameters	Patients (n = 87)	Healthy Controls (n = 87)	*P*
Male	50 (57.5)	50 (57.5)	.99
Female	37 (42.5)	37 (42.5)	
Age	49.7 ± 15.4	47.1 ± 14.6	.24
WBC (10^3^/UL)	13.39 ± 6.07	7.38 ± 1.54	**<.001**
NLR	8.86 ± 6.64	2.02 ± 0.97	**<.001**
HGB (g/dL)	13.79 ± 2.11	13.71 ± 2.19	.79
HCT (%)	40.87 ± 5.18	41.03 ± 6.11	.86
PLT (10^3^/UL)	247.18 ± 82.69	259.75 ± 71.21	.30
PLR	238.11 ± 115.78	119.02 ± 65.38	**.02**
CRP (mg/L)	148.06 ± 114.25	3.61± 1.14	**<.001**
Albumin (g/dL)	3.57 ± 0.59	5.01 ± 3.63	**.005**
CRP/albumin ratio	46.42 ± 29.53	0.75 ± 0.42	**<.001**
Calcium (mg/dL)	8.73 ± 0.68	9.16 ± 0.53	**<.001**
Glucose (mg/dL)	166.32 ± 99.91	118.88 ± 45.71	**<.001**
AST (U/L)	93.17 ± 55.01	25.96 ± 14.47	**<.001**
ALT (U/L)	80. 51 ± 49.19	32.41 ± 29.51	**.003**
LDH (U/L)	312.63 ± 155.39	196.49 ± 54.61	**<.001**
Creatinine (mg/dL)	0.79 ± 0.47	0.85 ± 0.42	.35
Urea (mg/dL)	29.69 ± 17.19	29.81 ± 10.97	.96
GFR (mL/min)	98.16 ± 24.88	98.64 ± 23.05	.90
Sodium (mEq/L)	137.01 ± 3.79	139.34 ± 2.57	**<.001**
Potassium (mEq/L)	4.12 ± 0.56	4.39 ± 0.39	**<.001**
Triglyceride (mg/dL)	365.28 ± 155.66	166.51 ± 110.28	**.01**
LDL-C (mg/dL)	91.03 ± 39.18	105.35 ± 30.01	**.01**

Student’s *t*-test.

WBC, White blood cells; NLR, Neutrophil to lymphocyte ratio; HGB, Hemoglobin; HCT, Hematocrit; PLT, Platelet; PLR, Platelet-to-lymphocyte ratio; CRP, C-reactive protein; AST, Aspartate aminotransferase; ALT, Alanine aminotransferase; LDH, Lactate dehydrogenase; GFR, Glomerular filtration rate; LDL-C, Low density cholesterol.

**Table 4.** Changes in H19 and MALAT1 Cycle Threshold Values in Patient and Healthy Groups (n = 174)

Parameters	Healthy Controls (n = 87)				Patients (n = 87)			
	Mean	SD	Max.	Min.	Mean	SD	Max.	Min.
**H19 Ct**	−0.398	0.508	7.795	−8.140	−0.092	0.318	7.875	−4.830
**MALAT1 Ct**	−3.119	0.350	3.700	−10.035	−2.098	0.245	3.905	−6.935

Student’s *t*-test.

Ct, cycle threshold; SD, standard deviation.

**Table 5.** H19 and MALAT1 Difference Tests Between Healthy and Patient Groups

	T Statistic	Degrees of Freedom	Average Difference	Lower Confidence Limit	Upper Confidence Limit	*P*
H19	−0.509	144.324	−0.305	−1.490	0.880	.619
MALAT1	−2.383	154.020	−1.021	−1.867	−0.174	.018*

Student’s *t*-test.

MALAT1, Metastasis-associated lung adenocarcinoma transcript 1.

**Table 6.** Assessment of Relationships Between H19, MALAT1, and Other Measurements (n = 87)

Parameters	H19		MALAT	
	*r*	*P*	*r*	*P*
CRP (mg/L)	0.06	.45	0.13	.11
PCT (ng/mL)	0.19	.18	0.003	.99
WBC (10^3^/UL)	0.03	.74	0.07	.34
NLR	0.03	.70	0.02	.79
PLR	0.09	.22	−0.01	.89
CRP/albumin ratio	0.05	.54	0.12	.13
Lactate (mmol/L)	0.18	.15	−0.09	.46
Hospitalization (days)	−0.15	.18	−0.04	.71
RANSON at Hospitalization	0.14	.21	0.12	.26
RANSON 48th Hour Score	0.13	.24	−0.02	.87
BISAP Score	−0.09	.44	0.05	.67
BMI kg/m^2^	0.12	.26	0.01	.94

Pearson correlation.

CRP, C-reactive protein; PCT, Procalcitonin; WBC, White blood cells; NLR, Neutrophil to lymphocyte ratio; PLR, Platelet-to-lymphocyte ratio; BMI, Body mass index.

**Table 7.** Assessment of H19 and MALAT1 in View of Need for ICU Admission (n = 87)

	H19	MALAT1
Intensive care unit need No (n = 83) Yes (n = 4)	0.04 ± 0.94−2.94 ± 1.94	−2.06 ± 1.31−2.94 ± 1.08
*P*	**.049**	.47

Student’s *t-*test.

## Data Availability

The data that support the findings of this study are available on request from the corresponding author.
